# Identification of Na^+^/K^+^-ATPase Inhibitor Bufalin as a Novel Pseudorabies Virus Infection Inhibitor In Vitro and In Vivo

**DOI:** 10.3390/ijms241914479

**Published:** 2023-09-23

**Authors:** Zongyi Bo, Jinjin Zhu, Xiaojuan Li, Chengcheng Zhang, Mengjiao Guo, Yongzhong Cao, Xiaorong Zhang, Yantao Wu

**Affiliations:** 1 Joint International Research Laboratory of Agriculture and Agri-Product Safety, The Ministry of Education of China, Yangzhou University, Yangzhou 225009, China; zybo@yzu.edu.cn (Z.B.);; 2 College of Veterinary Medicine, Yangzhou University, Jiangsu Co-Innovation Center for the Prevention and Control of Important Animal Infectious Disease and Zoonoses, Yangzhou 225009, China

**Keywords:** pseudorabies virus, natural compound, bufalin, therapeutic effect

## Abstract

Pseudorabies virus (PRV), an alpha herpesvirus, induces significant economic losses to the swine industry and infects multiple kinds of animals. Therefore, it is of great importance to explore anti-PRV compounds. In this study, to explore the anti-PRV compounds, a library of natural compounds was screened through a cell-based ELISA assay, and it was discovered that bufalin, a Na^+^/K^+^-ATPase inhibitor, had a robust inhibitory effect on PRV replication. A time-of-addition experiment and temperature-shift assay showed that bufalin significantly inhibited the entry stage of PRV. NaCl- or KCl-treatment showed that NaCl could enhance the inhibitory effect of bufalin on PRV replication, whereas there was no significant effect under the treatment of KCl. Meanwhile, it was also found that bufalin possessed antiviral activity against other alpha herpesviruses, including human herpes simplex virus type 1 (HSV-1) and chicken Marek’s disease virus (MDV). Finally, it was found that bufalin could decrease the viral load in multiple tissues, and reduce the morbidity and mortality in PRV-challenged BALB/c mice. Overall, our findings demonstrated that bufalin has the potential to be developed as an anti-PRV compound.

## 1. Introduction

Pseudorabies (PR) is an important disease in the swine industry caused by pseudorabies virus (PRV), which is also known as Aujeszky’s disease virus (ADV) because it was first discovered by Aujeszky in Hungary in 1902 [1]. PRV is a member of the herpesviridae family, which has double-stranded enveloped linear DNA [2]. The clinical signs caused by PRV differ depending on the stages of pigs. PRV can cause neurological symptoms and diarrhea symptoms in piglets, respiratory symptoms in large- and medium-sized pigs, and abortion and stillbirth in pregnant sows [3]. Pigs are the natural host of PRV, but it can also infect a variety of domestic and wild animals, causing fever, itching, encephalomyelitis, and 100% death in other animals [4]. Multiple reports have showed that PRV can infect not only animals but also human beings. The main clinical signs in human beings are encephalitis, swelling, pruritis, pain, sweating, fever, dysphagia, and aphthous stomatitis [5,6]. Therefore, it is of great value to develop anti-PRV drugs not only for animals but also for human beings.

Currently, the primary method for controlling PRV is the use of vaccines, including inactivated and live attenuated vaccines [7]. Among these vaccines, Bartha-K61 live attenuated vaccine is the most widely used PRV vaccine in the world, and it has shown a considerable protective effect against most prevalent PRV strains [8]. However, since 2011, PRV variants have emerged and spread rapidly in China, causing substantial economic losses to the Chinese swine industry [9,10,11]. A phylogenetic analysis showed that the newly happened PRV variants had a large genetic diversity with the classical strains. Additionally, PRV variants are more pathogenic to pigs compared with the classical virulent PRV strains [12,13]. Multiple studies have demonstrated that the Bartha-K61 vaccine cannot provide complete protection against the PRV variants [14]. Therefore, there is a need to develop and design effective anti-PRV drugs to improve the prevention and therapeutic strategies against PRV infection.

Natural products have a long history in antivirus research, as they have shown efficient therapeutic activity against multiple kinds of viruses [15]. In comparison to conventional antiviral drugs, natural compounds have a minimal undesirable side effect and a lower risk of resistance. Many kinds of natural plants, fungi, bacteria, and animal-derived products have displayed an effective inhibitory effect to multiple kinds of herpesviruses. It has been discovered that honokiol can inhibit the replication of herpes simplex virus-1 (HSV-1) by inhibiting its DNA replication and gene expression [16]. In addition, it was found that artesunate derivative TF27 could inhibit the replication of Marek’s disease virus (MDV) and suppressed the tumor development induced by it [17]. Multiple natural compounds have shown anti-PRV capability, including (-)-Epigallocatechin-3-gallate (EGCG), resveratrol, quercetin, and isobavachalcone [18,19,20,21]. All these inhibitory compounds showed different anti-PRV mechanisms, including adsorption, entry, gene transcription and expression, and cell-to-cell spread.

In this study, we attempted to find the natural compounds that can inhibit PRV replication in vitro and in vivo. The findings suggested that bufalin had a significant inhibitory effect on PRV replication by inhibiting the entry stage of the PRV life. Moreover, it was found that bufalin had a broad antiviral activity against other alpha herpesviruses such as HSV-1, and MDV. Finally, it was demonstrated that bufalin could significantly decrease the viral load in multiple tissues, and it displayed good therapeutic activity by increasing the survival rate against PRV infection in mice. Taken together, this study highlights the significant inhibitory effect of bufalin against PRV infection in vitro and in vivo, providing a potential therapeutic strategy for the treatment of PRV infection.

## 2. Results

### 2.1. Screening of the Anti-PRV Compounds Using Cell-Based ELISA

**Figure 1:**
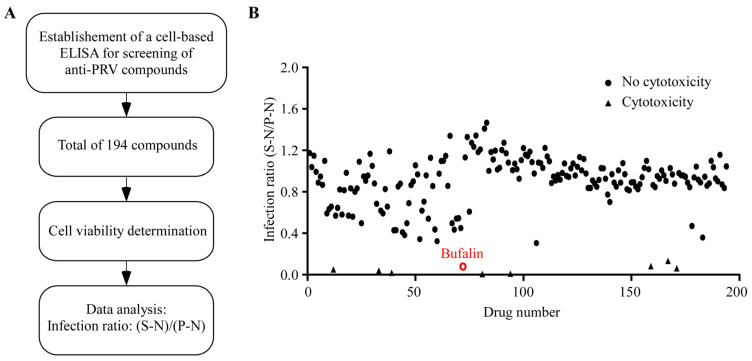
Processes of the anti-PRV compound screening and the effects of 194 natural products to the proliferation of PRV. (**A**) The processes of the anti-PRV compound screening. (**B**) The results of the inhibition ratio of different compounds to the replication of PRV. Briefly, PK-15 cells in 96-well plates were pre-treated with different compounds at a concentration of 10 μM for 2 h, and the cells were then infected with PRV JSY13 (0.15 MOI) for 24 h. Afterwards, the cells were then incubated with PRV-UL42 polyclonal antibodies as the primary antibody and goat anti-mouse IgG antibodies as secondary antibody. TMB substrate and stop solution were added and the values were read at OD_450_. Finally, the inhibition ratio was plotted as the percentage of PRV infection in compound-treated group compared to PRV infection in DMSO-treated control group.

Natural products have long been used as potential antiviral drugs for combating viruses. To screen the effective natural products that could inhibit the replication of PRV, a cell-based ELISA method was constructed due to its high throughput and accuracy advantages. The main processes of compound screening in this study are presented in Figure 1A. In brief, a compound library consisting of 194 natural products (Table S1) at a concentration of 10 μM was screened using the constructed cell-based ELISA method. The compound with cytotoxicity was eliminated and the inhibition ratio of each compound was calculated. The results showed that bufalin exhibited the highest inhibition ratio of over 90% among the 194 screened compounds (Figure 1B).

### 2.2. Bufalin Inhibited PRV Infection in a Dose-Dependent Manner

**Figure 2:**
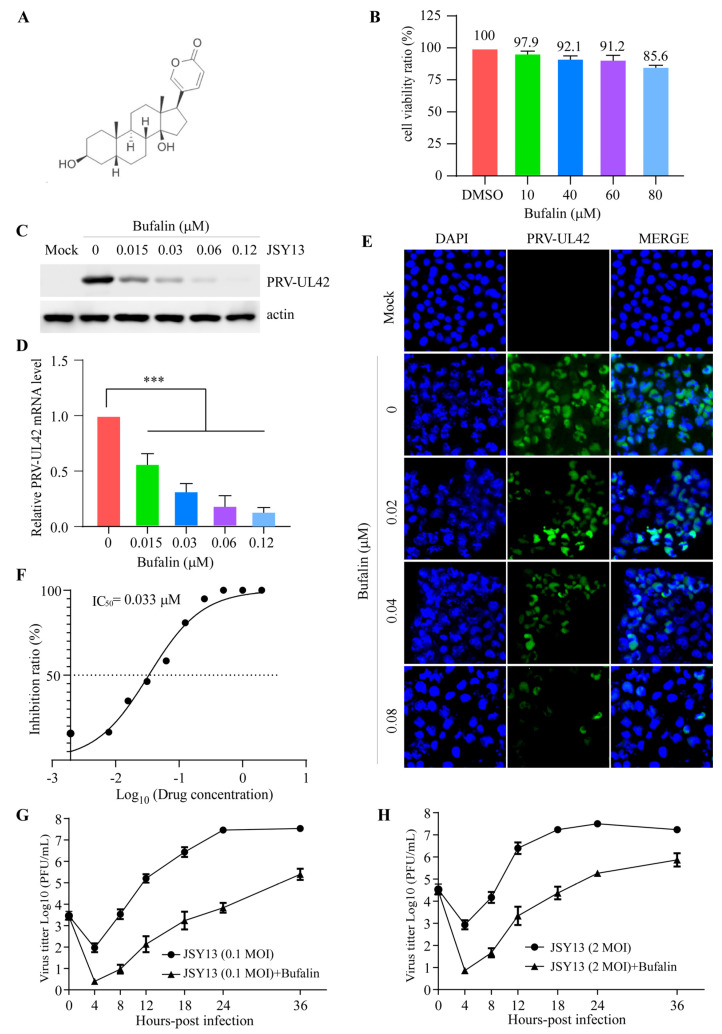
Bufalin inhibited the replication of PRV in PK-15 cells. (**A**) The structure of bufalin. (**B**) PK-15 cells in 96-well plates were treated with bufalin in concentrations of 10 μM, 40 μM, 60 μM, and 80 μM, respectively. Twenty-four hours later, the cells’ viability was detected using CCK-8 kit. (**C**) PK-15 cells in 6-well plates were pretreated with bufalin with concentrations of 0.015 μM, 0.03 μM, 0.06 μM, and 0.12 μM, respectively. Two hours later, the cells were infected with JSY13 (0.15 MOI) with bufalin in culture medium. Twenty-four hours later, the protein level of PRV-UL42 was measured using Western blot. (**D**) The experiments were performed as in (**C**), except the mRNA level of PRV-UL42 was measured using qRT-PCR. (**E**) PK-15 cells on the coverslips in 6-well plates were pretreated with bufalin with concentrations of 0.02 μM, 0.04 μM, and 0.08 μM, respectively. Two hours later, the cells were infected with JSY13 (0.15 MOI) for 18 h with bufalin in culture medium. The cells were stained with PRV-UL42 polyclonal antibodies (Green) and the nuclei were stained with DAPI (Blue). (**F**) PK-15 cells in 96-well plates were pretreated with bufalin with concentrations of 0.0039 μM, 0.0078125 μM, 0.015625 μM, 0.03125 μM, 0.0625 μM, 0.125 μM, 0.25 μM, 0.5 μM, 1 μM, and 2 μM, respectively. Two hours later, the cells were infected with JSY13 (0.15 MOI) with bufalin in culture medium. Twenty-four hours later, cell-based ELISA experiment was performed. (**G**,**H**) The PK-15 cells were pre-treated with bufalin (2 μM) or not, then the cells were infected with JSY13 at an MOI of 0.1 (**G**) or 2 (**H**), respectively. The cells were collected at 4, 8, 12, 18, 24, and 36 h post-infection; the viral titter was measured using plaque formation assay. (***, *p* < 0.001).

Bufalin is a type of steroid with a structure exhibited in Figure 2A. Before confirming the activity of bufalin against PRV infection, the cytotoxicity of it on PK-15 cells was first investigated. The PK-15 cells were treated with different concentrations of bufalin for 24 h and the cell viability was determined with CCK-8 reagents. The results showed that the cell viability was over 90% when the concentration was below 60 μM (Figure 2B). To confirm the inhibitory effect of bufalin on the replication of PRV, the PK-15 cells were pretreated with different concentrations of bufalin. The cells were infected with JSY13 at an MOI of 0.15 with bufalin in a culture medium and incubated for 24 h. The cells were collected and the Western blot experiment showed that bufalin inhibited the replication of PRV (Figure 2C). Meanwhile, the same results were shown in qRT-PCR (Figure 2D) and IFA experiments (Figure 2E) under the same conditions. Finally, a cell-based ELISA was performed to evaluate the IC_50_ of bufalin to PRV replication; the results showed that the IC_50_ of bufalin to the replication of PRV was as low as 0.033 μM (Figure 2F). Finally, the growth curve of the JSY13 on PK-15 cells with a 0.1 MOI and 2 MOI was created with bufalin treatment or not, respectively. The results showed that bufalin could inhibit the replication of PRV in both low MOI infection (Figure 2G) and high MOI infection (Figure 2H). All these data indicated that bufalin could significantly inhibit the replication of PRV.

### 2.3. Bufalin Inhibited PRV Infection by Interfering with Viral Entry

**Figure 3:**
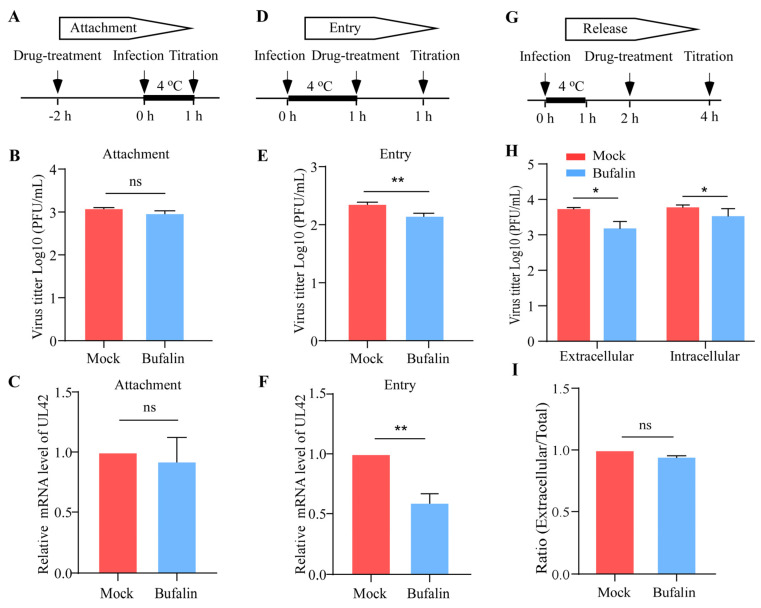
Bufalin interfered with the entry stage of PRV replication cycle. (**A**) The flowchart of viral attachment assay. (**B**,**C**) PK-15 cells were pretreated with bufalin (2 μM) at 37 °C for 2 h, then the cells were infected with JSY13 (5 MOI) with bufalin in culture medium and incubated at 4 °C for 1 h. Then, the viruses in cells were harvested and detected using plaque formation assay (**B**) and qRT-PCR (**C**). (**D**) The flowchart of viral entry assay. (**E**,**F**) PK-15 cells were infected with JSY13 (5 MOI) at 4 °C for 1 h, then the cells were treated with bufalin (2 μM) at 37 °C for 1 h. The cells were washed with sodium citrate (pH = 3) and the viral number in the cells was measured using plaque formation assay (**E**) and qRT-PCR (**F**). (**G**) The flowchart of viral release assay. (**H**) PK-15 cells were infected with JSY13 (5 MOI) at 4 °C for 1 h, then the culture medium was changed into fresh DMEM (2% FBS) and incubated at 37 °C for 2 h. Then, the cells were treated with bufalin (2 μM) at 37 °C for 1 h. The viral titer in supernatants and cells was tittered using plaque formation assay, respectively. (**I**) The ratio of the extracellular to the total number (extracellular + intracellular) of PRV. (ns, not significant; *, *p* < 0.05 and **, *p* < 0.01).

This study employed the time-of-addition and temperature-shift assays to investigate at which stage in PRV replication cycles that bufalin works. In the viral attachment assay (Figure 3A), the results show that the number of PRV in the bufalin-treated cells was not significantly different from that of the control group in either the plaque formation assay (Figure 3B) or the qRT-PCR (Figure 3C). In the viral entry assay (Figure 3D), the results show that the number of PRV was significantly inhibited using bufalin in the plaque formation assay (Figure 3E) and qRT-PCR (Figure 3F). In the viral release assay (Figure 3G), the results show that the viral titers in both the supernatants and cells of the bufalin-treated group were reduced compared with the control group (Figure 3H).However, the release ratio (extracellular/total) showed that bufalin had no significant effect on the release of PRV (Figure 3I). Taken together, these data demonstrated that bufalin could inhibit the entry of PRV, but had no effect on its attachment and release stage.

### 2.4. Na^+^/K^+^-ATPase Inhibitor Activity of Bufalin Was Involved in PRV Replication Suppression

**Figure 4:**
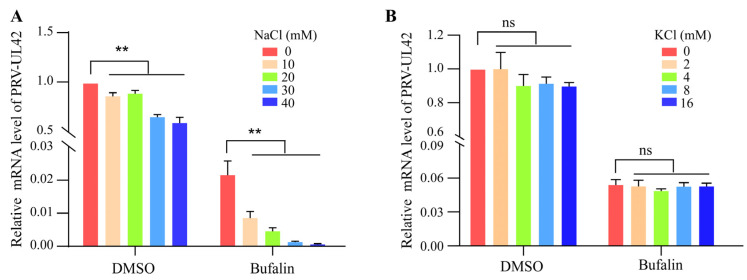
Bufalin inhibited the replication of PRV through Na^+^/K^+^-ATPase. (**A**) PK-15 cells in 12-well plates were pretreated with bufalin (2 μM) that contained increasing amounts of NaCl (0, 10, 20, 30, and 40 mM) for 2 h. Then, the cells were infected with JSY13 (0.15 MOI) and incubated for 24 h. The cells were collected using Trizol, and then total RNA was extracted and reverse transcribed into cDNA. Finally, the mRNA level of PRV was evaluated with qRT-PCR. (**B**) The experiments were performed as in (**A**), except the cells were pretreated with increasing amounts of KCl (0, 2, 4, 8, and 16 mM). (ns, not significant; **, *p* < 0.01).

Like other cardiac glycosides, bufalin also possesses notable Na^+^/K^+^-ATPase inhibitor activity [22]. To determine whether Na^+^/K^+^-ATPase inhibitor activity of bufalin was involved in the suppression of PRV replication, PK-15 cells were pretreated with bufalin containing various concentrations of NaCl or KCl for 2 h. Then, the cells were infected with JSY13 and incubated for 24 h. The PRV in cells was collected and the mRNA level was evaluated using qRT-PCR. The results showed that PRV replication was significantly inhibited with the increased NaCl dosage compared with the control treatment. Moreover, the addition of NaCl increased the inhibitory effect of bufalin on the replication of PRV (Figure 4A). In contrast, the replication of PRV had no significant difference with the increasing amount of KCl, neither in normal nor bufalin-treated conditions (Figure 4B). These data demonstrated that the concentration of Na^+^ had a significant role in PRV replication, whereas the concentration of K^+^ had no significant effect on PRV replication.

### 2.5. Bufalin Showed a Wide Inhibitory Spectrum to Other Herpesviruses

**Figure 5:**
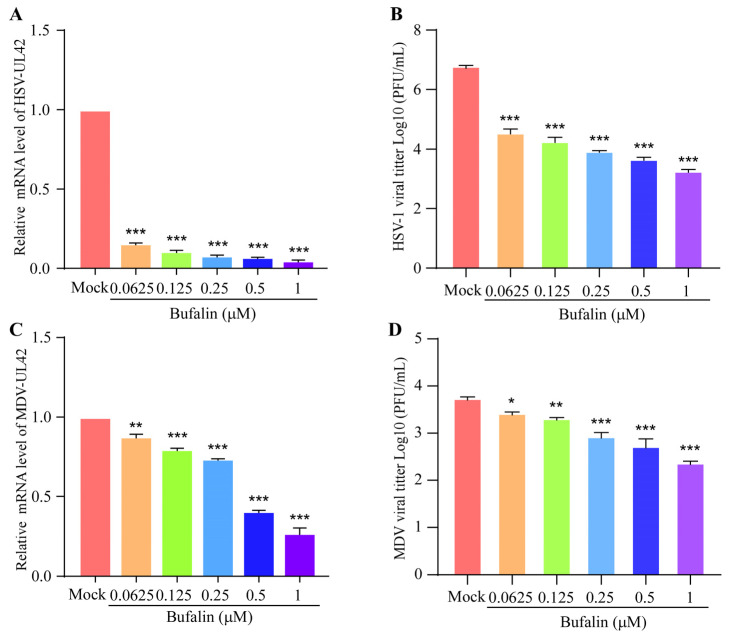
Bufalin inhibited the replication of HSV-1 and MDV. Vero cells in 6-well plates were pretreated with bufalin in concentrations of 0.0625 μM, 0.125 μM, 0.25 μM, 0.5 μM, and 1 μM, respectively. Two hours later, the cells were infected with HSV-1 (0.1 MOI) for 24 h. The cells were collected and the mRNA level of HSV-UL42 was measured using qRT-PCR (**A**), or the viral titer was measured using plaque formation assay (**B**). The CEF cells were pretreated with bufalin in concentrations of 0.0625 μM, 0.125 μM, 0.25 μM, 0.5 μM, and 1 μM, respectively. Two hours later, the cells were infected with MDV JS2018 strain (0.02 MOI) for 48 h. The cells were collected and the mRNA level of MDV-UL42 was measured using qRT-PCR (**C**), or the viral titer was measured using plaque formation assay (**D**). (*, *p* < 0.05; **, *p* < 0.01; and ***, *p* < 0.001).

To determine whether bufalin has a broad antivirus activity, human herpes simplex virus type 1 (HSV-1) and avian Marek’s disease virus (MDV) were chosen for a further analysis. Vero cells were pretreated with increasing amounts of bufalin for 2 h, and then were infected with HSV for 24 h. The mRNA level of HSV-UL42 was detected, and the results showed that bufalin could significantly inhibit the replication of HSV in a dose-dependent manner (Figure 5A). A similar result was shown by measuring the HSV-1 viral titter using a plaque formation assay (Figure 5B). CEF cells were pretreated with an increasing amount of bufalin for 2 h, and then were infected with MDV for 48 h. The mRNA level of MDV-UL42 was measured, and the results showed that bufalin could significantly inhibit the replication of MDV (Figure 5C). A similar result was shown by measuring the MDV viral titter using a plaque formation assay (Figure 5D). Taken together, we found that bufalin had a broad inhibitory effect on the other alpha herpesviruses, including HSV-1 and MDV. Furthermore, the inhibitory effect of bufalin on HSV-1 was more pronounced than that to MDV.

### 2.6. Bufalin Showed a Therapeutic Effect on PRV Infection in Mice

**Figure 6:**
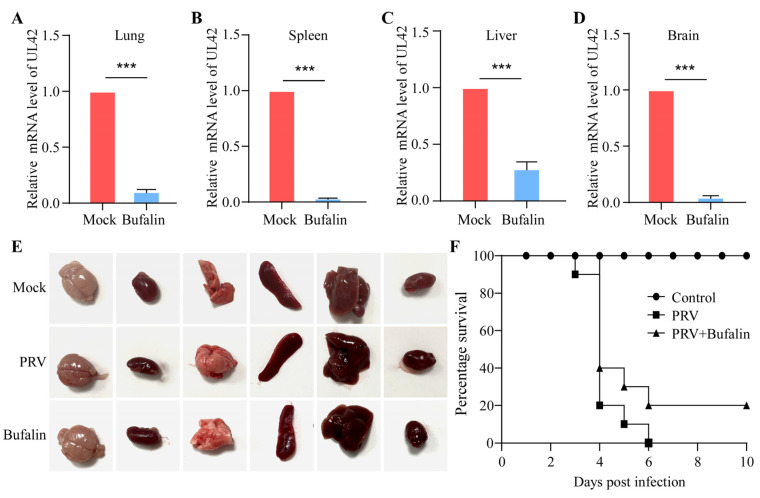
Bufalin inhibited the PRV infection in vivo. (**A**–**D**) The tissues were collected from each of three sacrificed mice at 2 days post-challenge in bufalin-mock-treated group or -treated group. Then, the RNA of them was extracted and trans-reversed into cDNA, and the mRNA level of PRV was detected in tissues of lung (**A**), spleen (**B**), liver (**C**), and brain (**D**). (**E**) The pathological examination of the sacrificed mice at 2 days post-challenge, and the typical figures, are presented. (**F**) The survival curve of mice in different groups. (***, *p* < 0.001).

As bufalin showed a significant inhibitory effect on the replication of PRV in vitro, we further checked whether bufalin could inhibit the PRV infection in a mice model. The mice were divided into three groups: a mock group, a JSY13-challenged group, and a bufalin-treated with JSY13-challenged group. The mice were injected intraperitoneally with 3000 PFU of JSY13, and bufalin was also injected intraperitoneally at a concentration of 0.5 mg/kg at every 36 h. The mice in the JSY13-challenged group displayed symptoms of lethargy, diarrhea, and scratching at around 48–60 h post-challenge, while three mice showed symptoms in the bufalin-treated group around 60 h. Three mice in each group were sacrificed at 48 h post-infection, and multiple tissues were picked for viral load detection. The viral load in the lung, spleen, liver, and brain was detected in both JSY13-challenged and bufalin-treated groups. The results showed that the viral load in all these tissues was significantly reduced in the bufalin-treated group (Figure 6A–D). In addition, pathological examination was performed by checking different organs of different groups; it was found that bufalin could reduce the hemorrhage and congestion signs in the brain induced by JSY13, while it seems not obvious in other organs (Figure 6E). Finally, the survival of each group was recorded, and all the mice in the JSY13-challenged group died within 6 days post-challenge. However, two mice survived in the bufalin-treated group, which indicated that bufalin increased the survival rate to 20% (2 out of 10 mice survived) (Figure 6F). Overall, the results demonstrated that bufalin could delay the onset of clinical symptoms, decrease the viral load in multiple tissues, and increase the survival rate of PRV-infected mice.

## 3. Discussion

Pseudorabies disease (PR) is typically controlled with vaccines in the global swine industry. However, outbreaks still occur due to the emergence of variant strains. PRV has caused significant economic losses to swine production. Previously, it was reported that there were 2600 newborn piglets and 200 sows that died from the PRV variant infections in Henan Province just in one herd [23]. In addition to the development of new PRV vaccines, exploring antiviral drugs that can inhibit PRV infection is also a potential solution for controlling pseudorabies. Furthermore, the investigation of anti-PRV compounds can also serve as a reference for the development of antiviral compounds for other herpesviruses. In this study, we found that bufalin showed a significant inhibitory effect on PRV infection both in vitro and in vivo, providing insight into possible therapeutic strategies for PRV infection.

In this study, a cell-based ELISA for anti-PRV compound screening was established, and the results showed that bufalin could significantly inhibit the replication of PRV. Bufalin, a cardiotonic steroid extracted from the skin and parotid venom glands of Chinese toad venom (*Bufo gargarizans*), is a traditional Chinese herb with a long history of anti-inflammasome and anticancer properties [24,25]. Until now, multiple biological activities of bufalin have been reported. It has been demonstrated that bufalin shows a significant anti-tumor effect against multiple tumors, such as leukemia and gastric, hepatocellular, and colorectal cancer, by inducing apoptosis, type I programmed cell death, or autophagy [26,27,28]. Additionally, bufalin was found to be an effective Na^+^/K^+^-ATPase inhibitor, which plays an important role in its anti-tumor activity [29,30]. Importantly, it has been reported that bufalin has an excellent antiviral acidity against both RNA and DNA viruses, including hepatitis B virus (HBV), porcine reproductive and respiratory syndrome virus (PRRSV), severe acute respiratory syndrome coronavirus 2 (SARS-CoV-2), Japanese Encephalitis Virus (JEV), and zika virus (ZIKV) [31,32,33,34,35]. Bufalin is one of the cardiac glycosides, which include drugs like ouabain, oleandrin, and digoxin that are a kind of drug that is typically used for the treatment of heart failure, atrial fibrillation, and atrial flutter [36]. Before confirming the inhibitory effect of bufalin on the replication of PRV, the cytotoxicity of it on PK-15 cells was determined. The results showed that bufalin had no obvious cytotoxic effect on PK-15 cells as the cell viability was still more than 90% when the concentration of bufalin was up to 60 μM (Figure 2B). Western blot, qRT-PCR, and IFA experiments were also performed, and the results showed that bufalin could significantly inhibit the replication (Figure 2C–E). Finally, the IC_50_ of bufalin for the replication of PRV was determined using the established cell-based ELISA assay, and the results showed that the IC_50_ was as low as 0.033 μM (Figure 2F), which indicated that bufalin had a strong inhibitory effect on the replication of PRV. All these data indicated that bufalin could be a potential treatment opinion for PRV infection.

Antiviral drugs have been reported to disrupt viral replication by targeting different stages of the viral life cycle, such as attachment, entry, and release stages [37,38,39]. In this study, we aimed to determine which stage in the PRV replication cycle bufalin works by performing time-of-addition and temperature-shift experiments. In experiments exploring the effect of bufalin on PRV attachment, the cells were pretreated with bufalin at 37 °C for 2 h, and then infected with JSY13 at 4 °C for 1 h. The plaque formation assay and qRT-PCR experiment showed that bufalin had no effect on the viral titter or PRV genomic RNA levels of PRV attached to PK-15 cells (Figure 3B,C). In experiments exploring the effect of bufalin on PRV entry, the cells were infected with JSY13 at 4 °C for 1 h and then treated with bufalin at 37 °C for 1 h. The results of the plaque formation assay and qRT-PCR experiment showed that bufalin could significantly decrease the viral titter and the PRV genomic RNA levels of viruses that entered into the PK-15 cells (Figure 3E,F). In experiments exploring the effect of bufalin on PRV release, the cells were infected with JSY13 at 4 °C for 1 h and then treated with bufalin at 4 °C for 1 h and an additional 1 h at 37 °C, followed by a 2 h treatment at 37 °C. The plaque formation assay was used to detect the viral titers extracellularly and intracellularly, and the ratio of extracellular to the total amount of viruses was calculated. The results showed that bufalin had no significant inhibitory effect on the release of PRV, though the viruses in extracellular and intracellular environments were slightly inhibited (Figure 3H,I). Combinedly, these data showed that bufalin could inhibit the replication of PRV by interfering with the entry stage.

Na^+^/K^+^-ATPase is an important target of bufalin. To demonstrate whether Na^+^/K^+^-ATPase was involved in the inhibitory effect of bufalin on the replication of PRV, PK-15 cells were pretreated with bufalin in the presence of different concentrations of NaCl or KCl before PRV infection. The viral genomic RNA levels were detected using qRT-PCR, and the results showed that a higher concentration of NaCl in the extracellular environment led to a significant inhibitory effect on PRV replication whether bufalin was added or not. However, the replication of PRV was not affected with the addition of KCl (Figure 4A,B). This phenomenon differed from other reports, which showed that KCl could recover the inhibitory effect of bufalin on SARS-CoV-2 and JEV [34,35]. Meanwhile, this study also demonstrated the broad antiviral spectrum of bufalin, as it could still inhibit the replication of HSV-1 in Vero cells and MDV in CEF cells (Figure 5A–D). As Na^+^/K^+^-ATPase was involved in multiple cellular signaling pathways, the specific cellular pathway and host cellular targets of the inhibitory effect of bufalin on the replication of PRV and other alpha herpesviruses warrant further investigation.

Furthermore, to investigate whether bufalin could inhibit the PRV infection in vivo, the mice were injected with bufalin via the i.p. route along with the challenge of PRV variant JSY13 strain. It was found that bufalin could decrease the viral load in multiple tissues, and increase the survival rate by up to 20%. Though the rate was not very high, as we know, the therapeutic efficiency of the compounds relies on the dose of challenged virus, dosage form, mode of administration, and pharmaceutic adjuvant. Therefore, our study just provides a potential compound that can be used in the future. Further experiments to comprehensively reveal the therapeutic effect of bufalin in vivo still need to be performed by evaluating the PRV viral load and the histopathological changes of different tissues.

In conclusion, our results showed that bufalin had a significant inhibitory effect on PRV replication by interfering with the entry stage in its replication cycle. Additionally, bufalin demonstrated broad antiviral activity to other herpesviruses, including HSV-1 and MDV. Finally, our study found that bufalin showed a positive therapeutic effect against a PRV challenge in mice. This study provides a potential antiviral compound for the treatment of pseudorabies.

## 4. Materials and Methods

### 4.1. Cells and Viruses

PK-15 cells and Vero cells were cultured in DMEM (Thermo Fisher Scientific, Waltham, MA, USA) containing 10% fetal bovine serum (LONSA SCIENCE SRL, Ciudad de la Costa, Uruguay) and 1% penicillin/streptomycin. Chicken embryo fibroblast (CEF) cells were prepared from 9-day-old specific-pathogen-free (SPF) chicken embryos and were cultured in medium 199 (Thermo Fisher Scientific, MA, USA) supplemented with 6% FBS and 1% penicillin/streptomycin. All cells were cultured in a 37 °C containing 5% CO_2_ incubator. The PRV JSY13 strain, MDV JS2018 strain, and HSV-1 F strain were stored in our lab.

### 4.2. Chemical Reagents and Antibodies

The natural compound library was purchased from Selleck (Houston, TX, USA). TMB substrates and DAPI were purchased from Eeyotime (Shanghai, China). An Enhanced Cell Counting Kit-8 (CCK-8) reagent was purchased from ApexBio (Houston, TX, USA). HRP- labeled goat anti-mouse IgG secondary antibodies and HRP-labeled goat anti-rabbit IgG antibodies were purchased from Proteintech (Wuhan, China). Alexa 488-conjugated goat anti-mouse IgG antibodies were purchased from Millipore (Billerica, MA, USA). PRV-UL42 mouse polyclonal antibodies was generated in our lab.

### 4.3. Immunoblotting Analysis

The immunoblotting analysis was performed as previously described with little modification [40]. In brief, PK-15 cells were pre-seeded in 6-well plates at a density of 6 × 10^5^ cells per well; the cells were then treated with different concentrations of bufalin for 2 h. The cells were infected with JSY13 at an MOI of 0.15 for 24 h. After being washed with PBS, the cells were collected using 2 × SDS buffer, subjected to SDS-PAGE, and transferred onto BioTrace NT Nitrocellulose (NC) membranes (Pall Corporation, Washington, DC, USA). The membranes were blocked in 5% skimmed milk and incubated with indicated antibodies overnight at 4 °C. After being washed with TBST, the membranes were incubated with respective secondary antibodies at 4 °C for 4 h. The bands were visualized using a Tanon 5200 system (Tanon, Shanghai, China).

### 4.4. Cell Viability Assay

The viability of PK-15 cells after bufalin treatment was determined using the CCK-8 reagent as previously described [41]. PK-15 cells were seeded in 96-well plates (2 × 10^4^ cells/well) and incubated at 37 °C for 12 h. The culture medium was then removed and different concentrations of bufalin were added to the cells at 37 °C for 24 h. Then, 10 μL of the CCK-8 solution was added to each well and incubated at 37 °C for 2 h and the absorbance value was measured. The cell viability was calculated as the ratio of the bufalin-treated group to DMSO-treated group.

### 4.5. Cell-Based ELISA

The screening of the natural compound library and the IC_50_ determination was measured using the cell-based ELISA method as previously described [33]. PK-15 cells were plated in 96-well plates. The cells were then treated with a different compound at 37 °C for 2 h, and then infected with JSY13 at an MOI of 0.15 at 37 °C for 24 h. After being fixed (PBS containing 4% paraformaldehyde and 0.1% Triton X-100) and blocked (3% skim milk for 2 h), the cells were incubated with PRV-UL42 antibodies at 37 °C for 2 h. The cells were incubated with HRP-conjugated goat anti-mouse secondary antibodies at 37 °C for 1 h. Finally, TMB substrates were added and incubated at 37 °C for 15 min and stopped with H_2_SO_4_ (2 M). Finally, the absorbance value was read at 450 nm.

### 4.6. qRT-PCR

The qRT-PCR was performed as previously described with little modification [42]. Total RNA was extracted using an RNA extraction kit (CwBiotech, Suzhou, China) according to the manufacturer’s instructions. The RNA was then reverse transcribed into cDNA using the reverse transcription reagent EasyScript Reverse Transcriptase (M-MLV, RNaseH-) (Transgen, Beijing, China). The mRNA level of the viruses was evaluated by measuring the UL42 gene of them, respectively. Fluorescent qRT-PCR qMix (Vazyme, Nanjing, China) was used according to the manufacturer’s recommendations. Fold changes of the level of mRNA were determined using the 2^−ΔΔCT^ method. The primers used are provided in Table 1.

### 4.7. Indirect Immunofluorescence Assay

The indirect immunofluorescence assay was performed as previously described with little modifications [43]. PK-15 cells were seeded on coverslips in six-well plates at 5 × 10^5^ per well, and pretreated with bufalin for 2 h. The cells were infected with JSY13 at an MOI of 0.15 at 37 °C for 24 h. After the cells were fixed and blocked, they were incubated with PRV-UL42 polyclonal antibodies at 37 °C for 1 h. After being washed with PBS, the cells were incubated with FITC-conjugated goat anti-mouse IgG secondary antibodies at 37 °C for 30 min. The nuclei were stained with DAPI (Beyotime, Shanghai, China) and the cells were visualized with the light source for microscopes U-HGLGPS (Olympus, Tokyo, Japan).

### 4.8. Plaque Formation Assay

The plaque formation assay was used to determine the viral titer. In brief, the samples were diluted in a 10-fold series and used to infect monolayer cells on a 6-well plate for 1 h. After washing with PBS, DMEM containing 2% FBS and 1% low-melting-point agarose was added. When visible plaque was formed after approximately 3 days of incubation, the cells were stained with 1% crystal violet for 4–5 h. The plaques were then numbered to determine the viral titer.

### 4.9. The Inhibitory Effect of Bufalin on Different Stages of PRV Replication

For the attachment stage, PK-15 cells were seeded in 6-well plates (6 × 10^5^ cells/well); 12 h later, the cells were treated with bufalin (2 μM) for 2 h. The culture medium was then discarded, and the cells were infected with JSY13 (5 MOI) in the presence of bufalin (2 μM) at 4 °C for 1 h. The viral titer was measured using the plaque formation assay, and the mRNA level of PRV was detected using qRT-PCR.

For the entry stage, PK-15 cells were seeded in 6-well plates (6 × 10^5^ cells/well); 12 h later, the cells were infected with JSY13 (5 MOI) at 4 °C for 1 h. The cells were then treated with bufalin (2 μM) at 37 °C for 1 h. After being washed with sodium citrate (pH = 3) and PBS, the viral titer of the cells was measured using the plaque formation assay, and the mRNA level of PRV was measured using qRT-PCR.

For the release stage, PK-15 cells were seeded in 6-well plates at a density of 6 × 10^5^ cells per well. After 12 h, the cells were infected with JSY13 (5 MOI) at 4 °C for 1 h. After being washed with PBS, the DMEM containing 2% FBS was added to the cells for 2 h. Then, the cells were incubated with bufalin (2 μM) at 37 °C for 1 h. The viral titer in supernatants and cells was tittered using the plaque formation assay, respectively.

### 4.10. Sodium and Potassium Treatment Assay

PK-15 cells were pre-seeded in 12-well plates and incubated with bufalin that contained different concentrations of NaCl or KCl for 2 h. The cells were then infected with JSY13 (0.15 MOI) at 37 °C for 24 h. Finally, the mRNA level of PRV was evaluated using qRT-PCR.

### 4.11. The Effect of Bufalin on PRV Infection in BALB/C Mice

The in vivo experiment was performed as previously described [44]. In brief, a total of 39 female BALB/c mice (4–6 weeks old) were randomly divided into three groups: the control group, the PRV-infected group, and the PRV-infected with bufalin-treated group. For PRV infection, the mice were intraperitoneally injected with 3000 PFU of JSY13. Bufalin was intraperitoneally administered at a dose of 0.5 mg/kg of body weight every 36 h until all mice in the PRV-infected group had died. Additionally, three mice from each group were sacrificed at 2 days post-challenge, and tissue samples were collected from the lungs, spleen, liver, and brain to detect mRNA levels of PRV using qRT-PCR. The mice were monitored twice daily, and the number of deaths in each group was recorded.

### 4.12. Statistical Analysis

GraphPad Prism 8.0 (GraphPad software, CA, USA) was used for the statistical analyses. The data were expressed as the mean ± standard deviation and analyzed with Student’s *t* test between different groups from at least three independent repeats [45]. Significance in all figures is indicated as follows: *, *p* < 0.05; **, *p* < 0.01; and ***, *p* < 0.001.

## Figures and Tables

**Table 1:** The primer sequences for qRT-PCR.

Name	Sequence
PRV-UL42-F	5′-GACCGTCTTCAACGTCACCT-3′
PRV-UL42-R	5′-GCATGATGCAGTAGTCGTTG-3′
Pig-GAPDH-F	5′-CCTTCATTGACCTCCACTACA-3′
Pig-GAPDH-R	5′-GATGGCCTTTCCATTGATGAC-3′
HSV-UL42-F	5′-GACACGGCCCTAAAGAAACC-3′
HSV-UL42-R	5′-GGAGGTCGCGAAAGTAACAC-3′
Monkey-GAPDH-F	5′-GCCTCAAGATCGTCAGCAAC-3′
Monkey-GAPDH-R	5′-GGTCATGAGTCCTTCCACGA-3′
MDV-UL42-F	5′- AGCGCATCCATCATTTGTCC-3′
MDV-UL42-R	5′- ACATCACAAATCGTTCCGGC-3′
Chicken-GAPDH-F	5′-CTGTTGTTGACCTGACCTGC-3′
Chicken-GAPDH-R	5′-TCAAAGGTGGAGGAATGGCT-3′

## Data Availability

The data presented in this study are available on request from the corresponding author.

## Supplementary Materials

The following supporting information can be downloaded at: https://www.mdpi.com/article/10.3390/ijms241914479/s1.
